# Supplementary material for: Generative Chemistry Platform for Small Molecules Targeting RNA: A Case Study for Chemical Optimization
Source: Comput Struct Biotechnol J. 2026 Sep 10;35(1):0218. doi: 10.34133/csbj.0218 (PMC13558819; doi:10.34133/csbj.0218)
Supplement: Supplementary file 1 [file csbj.0218.f1.zip › FINAL CSBJ - GenAI for SMolsTargeting RNA - Supp Data.docx]

Generative Chemistry Platform for Small Molecules Targeting RNA: A Case Study for Chemical Optimization – Supplementary Data

*Timothy E. H. Allen,^1^ Susan M. Boyd,^2^ Maurinne Bonnet,^3^ Rabia T. Khan^1^**

*^1^Serna Bio*

*^2^CompChem Solutions Ltd*

*^3^University of Michigan, Biointerfaces Institute*

*E-mail: rabia@serna.bio

## Supplementary Data

- Figure S1 - Distributions of physicochemical properties for all compounds in the Serna Bio Dataset.
- Figure S2 - Distributions of physicochemical properties for all RNA binder compounds in the Serna Bio Dataset.
- Figure S3 - Distributions of physicochemical properties for all compounds in the R-BIND Dataset.

See also, as an XLSX

- Table S1 - Serna Bio Compounds – Min, Max, Median and Mean of physicochemical properties.
- Table S2 - Serna Bio Binders – Min, Max, Median and Mean of physicochemical properties.
- Table S3 - R-BIND – Min, Max, Median and Mean of physicochemical properties.

*Figure S1 - Distributions of physicochemical properties for all compounds in the Serna Bio Dataset.*

*Figure S2 - Distributions of physicochemical properties for all RNA binder compounds in the Serna Bio Dataset.*

*Figure S3 - Distributions of physicochemical properties for all compounds in the R-BIND Dataset.*
